# Combining Organoid Models with Next-Generation Sequencing to Reveal Tumor Heterogeneity and Predict Therapeutic Response in Breast Cancer

**DOI:** 10.1155/2022/9390912

**Published:** 2022-08-22

**Authors:** Yuhong Liu, Yixiang Gan, NiJiati AiErken, Wei Chen, Shiwei Zhang, Jie Ouyang, Leli Zeng, Di Tang

**Affiliations:** ^1^The Seventh Affiliated Hospital of Sun Yat-Sen University, General Surgery, Shenzhen 518107, China; ^2^School of Medicine, Sun Yat-Sen University, Shenzhen 518107, China; ^3^The Seventh Affiliated Hospital, Guangdong Provincial Key Laboratory of Digestive Cancer Research, Shenzhen 518107, China; ^4^Department of Breast Surgery, Dongguan Tungwah Hospital, Dongguan 518107, China

## Abstract

Estrogen receptor-positive (ER+) breast cancer (BC) is a common subtype of BC with a relatively good prognosis. However, recurrence and death from ER+ BC occur because of tumor heterogeneity. This study aimed to explore tumor heterogeneity using next-generation sequencing (NGS) and tumor-organoid models to promote BC precise therapy. We collected needle biopsy, surgical excision, and cerebrospinal fluid (CSF) samples to establish tumor organoids. We found that the histological characteristics of organoids were consistent with original lesions and recapitulated their heterogenicity. In addition, the NGS results showed that PIK3CA and TP53 genes had detrimental mutations. BAP1, RET, AXIN2, and PPP2R2A genes had mutations with unknown function. The score for homologous recombination deficiency (HRD) of genome was 56, indicating that the tumor was likely sensitive to PARPi. The mutant-allele tumor heterogeneity (MATH) value of the tumor genome was 68.03, indicating high tumor heterogeneity. At last, we performed a drug screening on organoids. The toxicity of different drugs toward BC organoids originated from needle biopsy and surgical excision was tested, respectively. The IC_50_ values in the needle biopsy groups were paclitaxel 2.83 *μ*M, carboplatin 61.47 *μ*M, neratinib 0.8 *μ*M, lapatinib >100 *μ*M; in the surgical excision groups: trastuzumab >100 *μ*M, docetaxel 0.036 *μ*M, tamoxifen 20.54 *μ*M, olaparib 5.478 *μ*M, BYL719 < 0.1 *μ*M. The toxicity data showed that the BC organoids could show dynamic characteristics of tumor progression and reflect the heterogeneity of BC. Our study demonstrates that the combined use of tumor organoids and NGS is a potential way to test tumor heterogeneity and predict drug response in ER + BC, which contributes to the development of personalized therapy.

## 1. Introduction

Breast cancer (BC) has surpassed lung cancer as the most commonly diagnosed cancer in women worldwide, killing almost 2 million females each year [1]. The most prevalent subtype of BC is estrogen receptor-positive (ER+) BC, accounting for more than 80% of cases [2]. Endocrine therapy utilizing well-defined indicators of hormone receptor expression has been established in recent years, which significantly increases the five-year survival rate of ER+ BC patients. While ER+ BC has a better prognosis than ER- BC, around 10% to 20% of ER+ BC patients still experience recurrence and metastasis [3]. Nearly all deaths from BC are associated with metastasis rather than the primary tumor, and brain metastasis is particularly a common feature of ER+ BC [4]. It is widely acknowledged that brain metastasis is an increasing problem for patients with metastatic BC and a cause of morbidity and mortality [5, 6].

The mechanism underlying inefficient ER+ BC therapy, recurrence, and metastasis is poorly understood. A possible account for this is genomic and intratumor heterogeneity. While BC is relatively less aggressive than many other solid tumors (e.g., liver cancer and pancreatic cancer), its high heterogeneity frequently results in tumor invasion, recurrence, metastasis, and therapy resistance [7]. Tumor heterogeneity can be divided into two categories: intertumor heterogeneity and intratumor heterogeneity. Intertumor heterogeneity, also known as tumor-to-tumor heterogeneity, refers to disparities between cancers from different patients, which complicate the search for a universal cure. However, even patients with identical tumor biomarkers tend to have varying therapeutic responses. Intratumor heterogeneity, which means heterogeneity within a single tumor, comprises phenotypic, epigenetic, genetic, histological, and behavioral diversities among subpopulations of cancer cells that provide fuel for drug resistance and disease progression [8, 9].

The rapid advancement of next-generation sequencing (NGS) technology has enabled researchers to decipher the heterogeneity of BC at the genomic level. Further refinement of intrinsic subtypes of gene expression profiles will significantly aid in developing personalized treatments for BC. Parker et al. have developed a 50-gene (PAM50) subtype predictor using microarray and quantitative reverse transcriptase-polymerase chain reaction (qRT-PCR) technology to stratify BC into gene expression-based intrinsic subtypes and provide outcome predictions in patients diagnosed with ER+ or ER- breast cancer [10]. The use of mRNA expression of PAM50 as a predictive marker for targeted therapy in a group of BC patients was described at the 2016 San Antonio Breast Cancer Conference [11]. It is expected that PAM50-based subtype could refine risk stratification and improve disease management [12]. Given that BRCA1 and BRCA2 (BRCA1/2) deficiency is a common mutation in BC and confers impaired homologous recombination repair (HRR) phenotype to tumor cells, poly ADP-ribose polymerase (PARP) inhibitors (PARPi) should be utilized as an example of precision medicine targeting DNA damage response [13]. In recent years, as high-throughput sequencing technology has advanced, British scientists have proposed ten new BC gene markers, which may lead to an improvement in risk assessment and personalized treatment [14]. The Fudan classification proposed by Shao et al. classifies triple-negative breast cancer (TNBC) into four transcriptome-based subtypes with different therapeutic targets or biomarkers [15]. In addition, NCCN guidelines suggest genetic expression assays, such as 21-gene and 70-gene testing, to guide customized diagnosis and treatment [16, 17]. However, in general, genetic testing results for prognosis, diagnosis, and treatment are not totally accurate. Tumor-associated biological changes are complicated, involving changes at different levels, from a monogenetic mutation to multiple up-/downstream pathway alterations.

An organoid is a preclinical model that simulates the biological and behavioral properties of primary tumors. In comparison to 2-dimensional (2D) cell culture, patient-derived organoids with 3-dimensional (3D) structures can capture the original heterogeneity and represent the complexity of the primary tumor [18, 19]. In 2018, Hans et al. established a living biobank of BC organoids [20]. In their study, the histopathological and genomic changes of the primary and metastatic BC organoids were consistent with the original tumors. Additionally, a recent study conducted on bladder cancer organoids demonstrated that organoids could retain the heterogeneity of the original tumor and recapitulate the dynamic evolution of the parental tumors in culture, presenting them as a reliable model system for studying drug response in the context of precision medicine [21].

In this study, the specimens of a 41-year-old Chinese woman who eventually died three months after surgery from brain-metastatic ER + BC were used. We hypothesized that the existence of intratumor heterogeneity is a possible factor for treatment failure and cancer metastasis. Tumor samples were successively obtained for sequencing and organoid culture. Organoids were used for drug screening. We report the use of tumor organoids combined with NGS for the investigation of intratumor heterogeneity and prediction of therapeutic responses for ER+ breast cancer patients.

## 2. Materials and Methods

### 2.1. Clinical Data and Patient Samples

The clinical information of the ER + metastatic BC cases was exhibited in Table 1. Tumor tissues were collected immediately after a needle biopsy, surgical resection, and cerebrospinal fluid (CSF) drainage. The solid tumor tissues were cut into two parts for histopathology evaluation and organoid generation. The liquid sample was collected in a sterile tube to isolate tumor cells. Extra tumor, normal adjacent tissues, and blood samples were collected for next-generation sequencing. This study was approved by the ethical committees of the Seventh Affiliated Hospital of Sun Yat-Sen University (Shenzhen, China). All the procedures were carried out in accordance with the Declaration of Helsinki.

### 2.2. Organoid Model

The solid tumor samples were preserved in cold DMEM (GIBCO), supplemented with 10% FBS (GIBCO), 1% antibiotics (MELUN), and carried to the laboratory within 20 minutes on ice. The tissue was washed and minced, and then digested in 1 mL BC organoid medium (BCM) containing 2 mg/ml collagenase (Sigma, Saint Louis, MO, USA) supplemented with 100 ul 10 mg/mL Dispase, 10 ul 10 mg/mL DNaseI, 0.1% Y-27632 inhibitor on a shaker at 37°C for 2–8 h. During digestion, pipetting was applied frequently. The digested mixture was stewed for residual tissue sedimentation and the supernatant was collected to prepare for centrifugation at 7000 rpm for 30–60 seconds. Cell precipitates were washed with 1 ml DMEM and then centrifuged again. After washing, the cell precipitates were resuspended in 10–20 *μ*l BCM, then mixed with 10 mg·ml^−1^ Matrigel (R&D, R&D Systems, USA), and seeded in a prewarmed 48-well plate (Corning) at 20 *μ*l drops. The suspension was solidified in a 37°C and 5% CO_2_ incubator for 20 min, and then 300 *μ*l BC organoid medium was added to each well and changed every 2-3 days.

Passage was performed every 7–14 days, depending on the growth status of cells. Droplets were scraped from the bottom of the plate with the pipette tips and absorbed into the 1.5 ml EP tube along with the medium. After rapid centrifugation of the mixture in an EP tube, the supernatant was removed with 1 ml TrypLE Express (Invitrogen, Carlsbad, CA, USA) added for digestion on a shaker at 37°C until the matrix gel was invisible. Additionally, minute quantities of TrypLE Express were added to the leftover droplets on the plate bottom for digestion. To terminate the enzymatic process, the digested cell combination was immediately centrifuged and the supernatant was collected and rinsed with DMEM. The next steps were implemented as described above. The number of visual organoids was amplified in a 1:2–1:3 ratio per passage.

### 2.3. Histopathology Evaluation

Tissues and organoids were fixed with 4% paraformaldehyde, then dried, and paraffin-embedded. Sections were cut for HE staining and immunohistochemistry (IHC). Primary antibodies utilized in this work comprised anti-ER (Abcam, ab16660, 1 : 300), anti-PR (Abcam, ab101688, 1 : 300), and anti-HER2 (Abcam, ab16662, 1 : 100). All images were obtained using an Olympus microscope (Olympus, Japan).

### 2.4. Drug Sensitivity Test

The vitality of organoids treated with drugs was determined using the CCK-8 assay. In brief, the dissociated organoids (3000 cells/well) were grown for 3 days in a 5% Matrigel precoated 96-well plate. On day 4, organoids were treated with drugs at various concentrations (0.1–100 uM). The CCK-8 solution was then added and incubated in a CO_2_ incubator for 4 hours. The light absorbance at 450 nm was determined using an Epoch multifunction microplate reader (Biotek, USA).

### 2.5. Next-Generation Sequencing and Data Analysis

Tissue samples were flash-frozen with liquid nitrogen and transported to −80°C for storage; organoid samples were scraped from the culture plate and washed three times with PBS. After centrifugation, the supernatant was collected, and the organoid precipitation was transported to −80°C for freezing. Using the DNeasy Blood and Tissue kit (QIAGEN), DNA samples were produced according to the manufacturer's instructions. The DNA products were validated using agarose gel electrophoresis equipment at a concentration of 0.8% (BOHAO). The Agilent SureSelect Human All Exon Kit (Agilent Technologies, CA, USA) was used to prepare libraries from qualified samples. The libraries were clustered utilizing Illumina's TruSeq PE Cluster Kit v4-cBot-HS on a cBot Cluster Generation System and sequenced with Illumina NovaSeq 6000. FastQC (v0.11.5) was utilized for DNA sequencing data quality assurance. Adapter trimming was accomplished using Trim Gale (v0.5.0). Sequence readings were mapped using Burrows–Wheeler Alignment with maximal exact matching (BWA-MEM)(v0.7.17) to the human reference genome GRCh38. MultiQC (v1.7) was used to collect and summarize QC information. SAM tools (v1.3.1) was used to sort alignment files and get data from BAM bam files. Genome Analysis ToolKit (v4.1.1.0) performed data preparation in accordance with best practice principles. By comparing each cancer sample to the reference blood leukocytes or nearby tissues, somatic mutations were identified. Using GATK(v3.6), mutations were evaluated. ANNOVAR (v160201) was utilized to annotate the result using dbSNP (v150), dbNSFP (v3.3a), COSMIC database (v70), and 1000 Genomes database (v201508). The R software was used for the HRD score and MATH value analysis.

### 2.6. Statistical Analysis

The dose-response curves and histogram of IC_50_ values were constructed using GraphPad Prism 8, and the findings were given as the mean and standard error of the mean. After obtaining IC_50_, the ranking location in the established organoids sensitivity database was used to analyze if the organoid was sensitive or resistant to a drug. More specifically, the IC_50_ value below the lowest 33.3% IC_50_ value was regarded as sensitive and higher than the top 33.3% was resistant. The middle part was regarded as unclear. The accuracy of the associations of drug sensitivity between organoid, NGS, and clinical outcomes was assessed. In this case, the clinical response of patients to pharmacological therapies was assessed using computed tomography (CT) or magnetic resonance imaging (MRI) in accordance with the Response Evaluation Criteria in Solid Tumors (RECIST) version 1.1. Partial response (PR) or complete response (CR) was labeled as sensitive, stable disease (SD) was labeled as unclear, and progressive disease (PD) was labeled as resistant.

## 3. Results

### 3.1. Patient's Information and Patient-Derived Organoids

**Figure 1:**
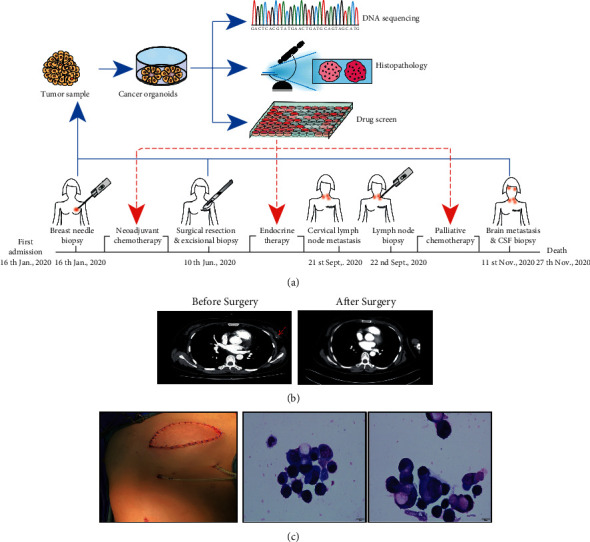
Diagnosis and treatment of metastatic ER+ breast cancer in a 41-year-old woman. (a) Diagnosis and treatment timeline. The blue arrow tips represent the gathering of tumor samples for the sequencing and cultivation of organoids. The red arrow tips denote the time points conducting organoids drug screen; (b) Tumor CT scan pictures.The red arrow denotes the tumor; (c) A gross chart displaying the surgical impact. Cells with irregular shapes are seen in CSF, indicating BC brain metastases. Scale bar, 10 μm.

The patient is a 41-year-old female who noticed a mass (4.0 × 3.9 cm) on her left breast and a mass (2.0 × 3.9 cm) on her left side of the axillary lymph node. She was diagnosed with invasive ductal carcinoma at stage III after a core needle biopsy. The patient achieved partial response on neoadjuvant chemotherapy (cyclophosphamide and doxorubicin) and was estimated to have stable disease after 3 weeks of docetaxel treatment. She then underwent modified radical mastectomy and adjuvant endocrine therapy (OFS + Tamoxifen). Three months after surgery, she developed cervical lymph node metastasis and showed resistance to endocrine therapy. The tumor progressed rapidly and PET-CT showed multiple metastases (lung, bone, and lymph nodes). Chemotherapy (cisplatin + vinorelbine, NP regimen) was administered for this change. However, the condition of the patient deteriorated quickly and finally, the patient died of breast cancer brain metastasis (Table 1 and Figure 1).

We collected the tumor samples from biopsy, surgical excision, and cells from the cerebrospinal fluid (CSF), and then generated organoids from primary and metastatic lesions. Finally, we successfully established organoids from three different specimens at various time points.

### 3.2. Histological Heterogeneity of Tumor-Organoid Pairs

**Figure 2:**
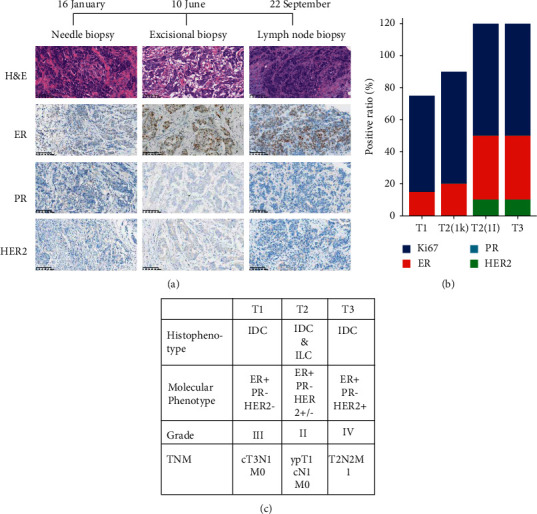
The patient tumors present a feature of histological heterogeneity. (a) Comparative H&E and immunohistochemical pictures of BC across time. Examples of ductal carcinoma (HER2-) from primary tumor biopsy (column on the left), ductal mixed with lobular carcinoma (HER2-/+) from surgical excision tumor (column on the middle), and ductal carcinoma (HER2+) from lymph node biopsy (column on the right) are displayed. Scale bar, 100 *μ*m. (b) The positive rate of immunohistochemistry markers changed throughout time. T1, time point 1, needle core biopsy; T2, time point 2, surgical excision. 1k and 1I, samples from separate tumor locations; T3, time point 3, cervical lymph node biopsy; (c) the table displays the frequency distribution of clinicopathological features at various time stages.

The histological heterogeneity of the tumor in the patient was revealed by means of histological and immunohistochemical staining. The characteristics of the primary tumor from needle biopsy samples were as follows: invasive ductal cancer, luminal B type, ER (15% weak +), progesterone receptor (PR) (-), androgen receptor (AR) (-), human epidermal growth factor receptor (HER2) (0), and Ki-67 (about 60% +). The histology characteristics of the surgical excision tumor samples were as follows: invasive ductal cancer mixed with invasive lobular carcinoma. The biomarker expression varied in different tumor region: specimen No.1I was ER (about 40% weak +), PR (-), HER2 (1 +), and Ki67 (approximately 70% +); specimen No. 1 K was ER (20% weak +), PR (-), HER2 (0), and Ki67 (70% +). The cervical lymph node metastasis was low-differentiated carcinoma and immunohistochemistry revealed the following: ER (about 40% weak medium +), PR (-), HER2 (1 +), and KI67 (approximately 70% +) (Figure 2). Conformably, counterpart patient-derived organoids (PDOs) have distinct histopathological characteristics.

**Figure 3:**
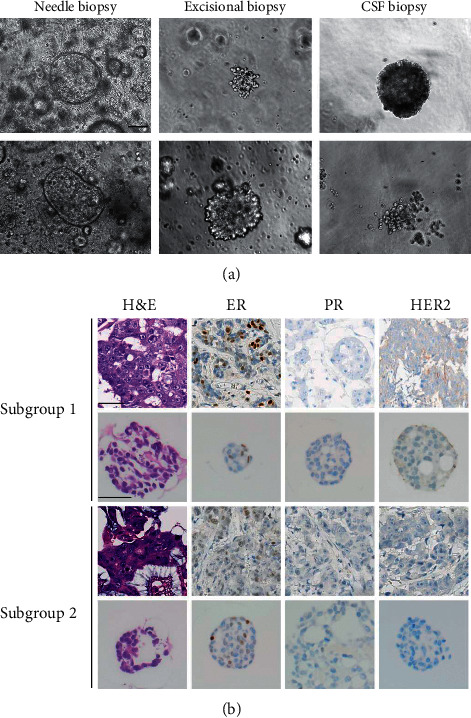
Breast cancer organoids recapitulate the histological heterogenicity of parental tumors. (a) Bright-field pictures illustrating the temporal change of BC organoid phenotypes. Shown are representative examples of cystic organoids from needle biopsy samples (column on the right), dis-cohesive organoids from surgery samples (middle top) and solid organoids from surgery samples (middle bottom), and dense solid organoids from CSF biopsy samples (right top) and grape-like organoids from CSF biopsy samples (right bottom). Scale bar, 100 μm.(b) Representative H&E and immunohistochemical staining photographs contrast the histology and receptor status of organoids subgroups with the spatial variation in the parent tumor. The ER, PR, and HER2 status of organoid lines generated from BCs is identical to the parent tumor. Scale bar, 50 μm.

Organoids were observed in a mixture of morphological types, including discohesive, cystic, and solid spherical. In addition, the proportion of each type shifted over time. Organoids generated from breast needle biopsy tended to be cystic and solid, while the proportion of discohesive type was increased in surgical excision tumor-derived organoids (Figure 3(a)). Subsequent investigation of marker expression demonstrated that organoids and paired primary tumors stained similarly for ER, PR, and HER2 (Figure 3(b)). Taken together, our findings show that organoids replicate the heterogenicity of histology and marker expression of tumor tissue.

### 3.3. Genomic Characterization of the Patient

#### 3.3.1. Variation of Single Genes

**Figure 4:**
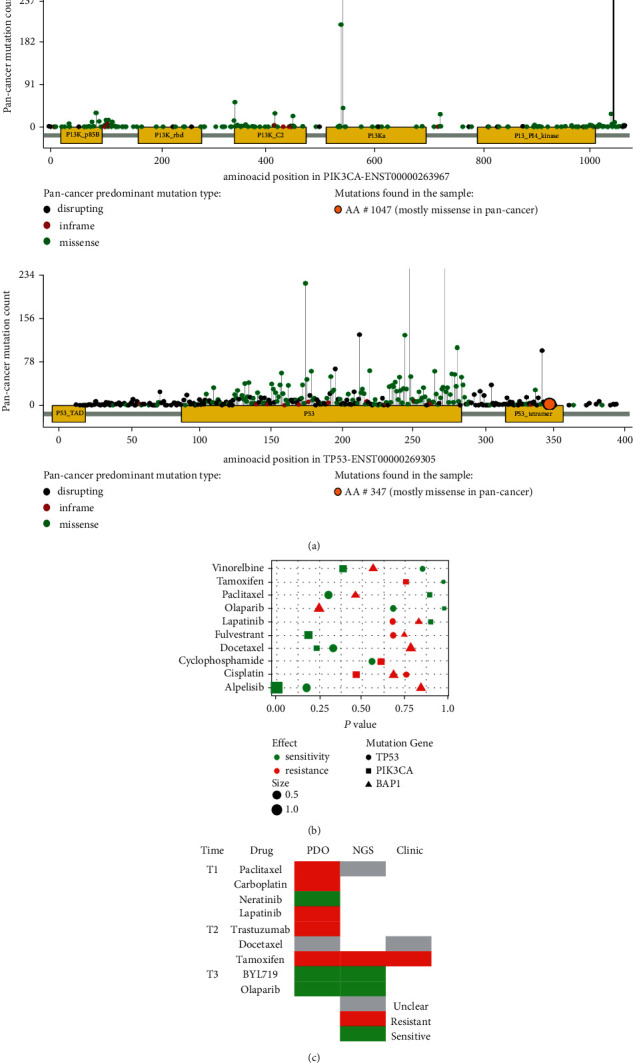
Breast cancer organoids reflect the clinical response and genetic prediction. (a) Variation landscape of the patient's PIK3CA and TP53 genes (46). (b) The gene mutation-related medication impact from the GDSC database is displayed as a scatter plot. (c) Assessment of PDOs, NGS-based prediction, and clinical response. The pharmacological responses for sensitivity, unclear, and resistance are colored in green, gray, and red, respectively.

This individual was determined to have a missense mutation H1047 R in exon 21 of the PIK3CA gene, which resulted in the substitution of histidine (H) for arginine (R) in amino acid 1047 of the PIK3CA protein's kinase domain (R). The H1047 R mutation increases the phosphorylation of downstream Akt and Mek1/2, allowing cells to survive in vitro without needing growth factors. Analysed by the Molecular Tumor Board Portal, PIK3CA bears somatic protein-affecting mutations in 35.4% of the invasive breast carcinoma samples (n=1297) and 14.0% of the pan-cancer samples (n=10703) [22] (Figure 4(a)).

Additionally, this individual was discovered to have a frameshift mutation in exon 10 of the TP53 gene, which caused the 348^th^ amino acid sequence to change from leucine (L) to tryptophan (W). Stop codons and the synthesis of truncated proteins in advance were predicted to have an effect on the function of TP53 protein. TP53 is a tumor suppressor gene involved in cell cycle arrest and apoptosis, and its loss of function resulted in the weakening or removal of the tumor suppressor. TP53 bears somatic protein-affecting mutations in 41.2% of the invasive breast carcinoma samples (n=1297) and 45.5% of the pan-cancer samples (n=10703) [22] (Figure 4(a)).

Nucleotides variants were also found in the AXIN2, BAP1, RET, PPP2R2A genes. According to a variety of computer analysis techniques and taking into account the subject's unique circumstances, the clinical significance of these mutations was considered to be clinically obscure (Table 2).

#### 3.3.2. Score for Homologous Recombination Deficiency (HRD)

We found that several genes were associated with homologous recombination repair (HRR) by means of pathway analysis. HRD score was calculated as the total score of loss of genome heterozygosity (LOH), telomeric allele imbalance (TAI), and transfer of large segments (LST) scores, which is HRD = LOH + TAI + LST. HRD score >42 is an indication of using PARP inhibitor for therapeutic trials (Table 3).

#### 3.3.3. Score for Mutant-Allele Tumor Heterogeneity (MATH)

MATH is an algorithm to quantify the genetic heterogeneity of tumor samples based on the mutation frequency of all alleles in the tumor. Through calculation, the MATH value can be obtained for each sample, and the MATH value reflects the level of tumor heterogeneity. The MATH score of this patient was 68.03 and the MATH value higher than 46 was judged to be highly heterogeneous [23]. Heterogeneity predicts a differential drug response. Meanwhile, studies showed that high MATH values correlated with breast cancer prognosis, especially in ER + positive breast cancer [24].

### 3.4. Drug Screening in Organoids

**Figure 5:**
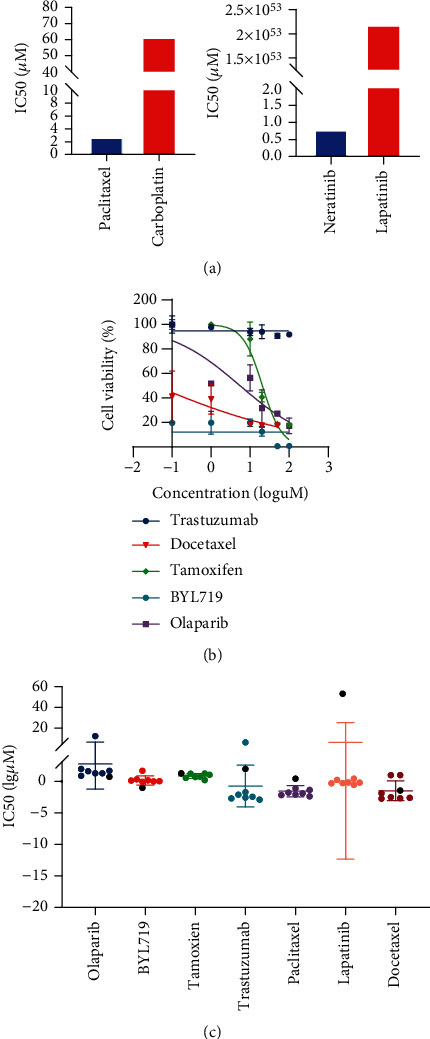
BC organoids allow in vitro drug screening (a) Bar graphs displaying the IC50 values of needle biopsy sample-derived organoids treated with paclitaxel, carboplatin, neratinib, and lapatinib; b) a representative image of dose response curves for surgery sample-derived organoids treated with trastuzumab, docetaxel, tamoxifen, BYL719, and olaparib. The error bars indicate the standard error of the mean for two to three separate experiments. (c) Scatter plot displaying the distribution of IC50 values of the medications in 8 organoids, with the patient's IC50 values shown in black.

Organoids were developed as substitutes for patients in drug testing. In terms of drug selection, we combined clinical therapy choices with extensive testing of NGS-predicted target medicines. The panel included cytotoxic drugs (paclitaxel, carboplatin, and docetaxel), EGFR/HER2 targeted drugs (lapatinib, neratinib, and trastuzumab), endocrine therapy drug (tamoxifen), PARPi (olaparib), and PIK3CA inhibitor (BYL719). Meanwhile, in order to coordinate the clinical practice as far as possible, we conducted drug susceptibility tests for several times. The IC_50_ values in the first test were paclitaxel 2.83 *μ*M, carboplatin 61.47 *μ*M, neratinib 0.8 *μ*M, and lapatinib >100 *μ*M; in the second test: trastuzumab >100 *μ*M, docetaxel 0.036 *μ*M, tamoxifen 20.54 *μ*M; in the third test: olaparib 5.478 *μ*M, BYL719 < 0.1 *μ*M (Figures 5(a) and 5(b)).

To begin with, we compared the organoids' drug test results to clinical responses. Although paclitaxel has a smaller IC_50_ than that of capecitabine, it belongs to the category of resistant drugs. For docetaxel, another taxane drug, the ranking of IC_50_ value indicated an unclear drug response in the test of organoids (Figure 5(c)). Actually, the patient achieved stable disease after using docetaxel, and according to the definition of the COSMIC database, the judgment of SD status was also unclear and controversial for drug efficacy, while this patient developed metastasis soon after treatment. In conclusion, taxane drugs are not the most effective chemotherapeutic agent for this patient; there may be primary or secondary resistance related to the patient's disease progression. As for tamoxifen, the endocrine therapy drug, the tumor progressed after two courses of treatment, suggesting the possibility of primary drug resistance, which agreed with our organoid drug test result. Moreover, the heterogeneity of HER2 expression were linked to the drug detection results of the HER2 family pathway drugs. It was found the organoids were resistant to the HER2 monoclonal antibody trastuzumab and the EGFR/HER2 dual-targeting agent lapatinib, but susceptible to the pan-targeting agent neratinib. Organoid susceptibility may aid in the selection of drugs targeting comparable targets or in the selection of follow-up therapies following the failure of first-line therapy. In addition, pharmacogenomics, in conjunction with PDOs drug susceptibility testing, was conducted.(Figure 4(b)).

We identified several gene mutations using next-generation sequencing, among which two were linked to treatment response. The PIK3CA mutation indicated that the tumor were susceptible to BYL719 but resistant to endocrine therapy [22]. With a high HRD rate (>42), tumors may be susceptible to PARPi. Both BYL719 and olaparib belonged to NGS-predicted sensitive drugs (Figure 4(c)).

## 4. Discussion

**Figure 6:**
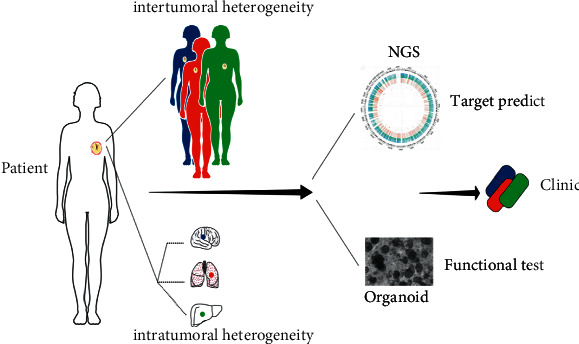
A potential integrated strategy for precision medicine of breast cancer treatment. Combined with next-generation sequencing, medication sensitivity might be directly evaluated using dose-response experiments on PDOs. In addition, both PDOs and tumors might be utilized to investigate the heterogeneity of tumors and assess the efficacy of target therapy.

In ER + BC patients, despite the overall effectiveness of antiendocrine treatment, primary and secondary drug resistance remains a major clinical problem. The considerable heterogeneity of BC, either interindividual and intratumoral, is frequently cited as an objective reason for therapeutic failure [7, 21, 25]. Here, we present a case of an ER+ BC patient with brain metastases three months after surgery. During her hospitalization, the imaging and pathological examinations confirmed that she was resistant to the chemotherapeutics and endocrine therapy suggested by the guidelines [17]. To verify the assumption of the existence of tumor heterogeneity and its role in therapeutic resistance, we established three organoid models from tumor cells obtained from different tumor specimens, i.e., needle biopsy, surgical excision, and CSF. What we attempted was to maximally mimic the complexity and heterogeneity of tumors and exploit a genome-based strategy of drug selection (Figure 6).

We confirmed that our models recapitulated the heterogeneity of the parental tumors. From the aspects of morphological observation, this case showed heterogeneous morphological characteristics, was notably distinct from previous breast cancer organoids, which may be a result of the primary intrinsic heterogeneity of tumors that developed from IDC into IDC mixed with ILC. This was also consistent with the previous study that ductal carcinomas often create solid, coherent organoids and lobular carcinomas produce mostly coherent organoids [20]. Actually, ductal carcinoma accounts for 75% of BCs, while lobular carcinoma accounts for less than 20% [26]. In metastatic breast cancer patients, the lobular tissue subtype represents an independent unfavorable prognostic risk. In a study of 13,111 individuals with metastatic breast cancer, ILC was associated with lower overall survival (OS) and progression-free survival (PFS) than IDC [27]. In terms of IHC typing, the expression of HER2 was altered with a transition from HER2- to HER2 1+ with tumor progression. Notably, in postoperative specimens, the 1I site was HER2 1+, the 1k site was HER2-, and all the lymph node metastases were HER2 1+. HER2 1+ or HER2 low expression is not classified as a subgroup in presented IHC molecular classification. However, BC with HER2 low expression has gained increasing interest [28]. Researchers aggregated data from four prospective clinical studies and found that the biology of HER2 low expression breast cancer tumors is distinct from that of HER2-breast cancer tumors [29]. In terms of pathological complete response (pCR) rates, patients with low HER2 expression had considerably lower pCR rates than those with HER2 IHC0 tumors, with the difference being even greater in ER-positive BC [29]. Due to the cross-reactivity between the ER and HER2 signaling pathways, decreased HER2 expression may operate as a treatment resistance factor in ER+ BC [30]. The heterogeneity of tumor histology may explain why this patient's therapy effect was worse than the majority of ER+ patients. In the established breast cancer organoids, we observed a similar degree of heterogeneity. Breast cancer organoids derived from postoperative specimens also performed differently in terms of IHC performance. The majority of the same subpopulation was detected as HER2-, but a few organoids stained faintly positive for HER2. Given that heterogeneity can be further classified into spatial and temporal heterogeneity, we found that both spatial and temporal heterogeneities serve as possible reasons for drug resistance and cancer metastasis via our models [8].

In recent years, as personalized therapy has emerged as a viable option for addressing disparities in drug responses, the investigation of BC heterogeneity has progressed from the histopathological and biobehavioral aspects to the molecular and genetic levels. The concept of “genetic inhomogeneity” within single tumors was first proposed in 1958 [31]. It has long been recognized that genetic variations play a vital role in cancer progression and the emergence of drug resistance. However, it is only with the advent of NGS technology that genomic landscapes of heterogeneous tumors and the underlying gene-drug associations can be adequately investigated [5, 31]. Given that targeted therapy must be based on identifying dominant drivers of oncogenesis, NGS should be a powerful implement for clinical decision-making and the development of biological agents [32]. For example, the application of NGS to studies of BRCA mutations in population-based cohorts has contributed to the update of clinical recommendations for disease screening and medication intervention, such as PARPi [33–35]. Despite the promising guiding significance of NGS, one problem is that NGS-detected alterations in gene signatures rely on the functional annotation to elucidate subsequent biological changes. In practice, most tumor mutations detected are classified as having uncertain functional consequences. The dynamic changes and interaction among genomic alterations create uncertainty in the predictive value. In the present study, the NGS sequencing revealed a high MATH score that indicated a high genetic heterogeneous. We innovatively combine NGS with drug screenings of cancer organoids generated from patient samples for functional verification. This is a novel strategy that might be more reliable than previous studies as NGS and organoid technology can be complementary [21, 36–39].

Organoids, as a recently developed preclinical model, can preserve key features and spatial architectures similar to that of the parental organs. Studies in multiple tumor organoids have evidenced that 3D organoid models can retain patient-specific physiological changes, including oxygen status, epigenetic and genetic marks, and gene-drug associations [37, 39–44]. In contrast to other personalized tumor models, like cancer cell lines and patient-derived xenografts (PDXs), organoids can be cultured from a small sample size (e.g., needle biopsy) and expanded with high efficiency from primary patient materials [19, 45]. Additionally, Ying's findings indicated a prediction specificity of 70%, a sensitivity of 83%, and an accuracy of 77% in the organoid drug test [46]. In our research, we made full use of samples of different stages of the disease and generated a series of organoid models to maximally mimic the spatial and temporal heterogeneity of the tumors, providing adequate reference data for drug selection. From this point, our research is a small step forward.

There are several limitations to this study. (1) This is a single case study. To raise the level of evidence, more studies are needed in the future, especially cohort studies. (2) Subject to economic, technical, ethical, and other factors, certain probably efficient drugs (e.g., BYL719) screened out by us were not applied to the patient, which constrained us from observing the drug responses in vivo. (3) NGS was merely conducted in tumor samples considering the patient's economic status and clinical practice. The genomic consistency between original tumors and organoids was not tested, although it has been validated by large quantities of studies. (4) The lack of immune system elements, other key stromal cells, and vasculature factors may hamper functional testing of drugs on organoid models.

## 5. Conclusions

In summary, the newly developed patient-derived organoid models can recapitulate the characteristics of parental tumors and reveal metastasis-related heterogeneity. It is feasible and promising to combine organoids with gene sequencing technology to aid in clinical decision-making.

## Figures and Tables

**Table 1:** Diagnosis and treatment process.

Date	Treatment	Tumor size, lymph nodes, distance transfer, and other data	Clinical response
01/22/2020 (01.16–01.23)	ddAC	One mass at 3 points on the left breast, about 4.0 *∗* 3.9 cm in size	ADM (NA)
		One mass on the left axillary lymph node, about 2.0 *∗* 3.0 cm in size	CTX (NA)
		No distant metastasis	
03/04/2020	ddAC	No palpable tumor on the left breast and the left axillary lymph node	ADM (PR)
			CTX (PR)
05/20/2020	ddT (The last time)	No palpable tumor on the left breast and the left axillary lymph node	DX (SD)
06/10/2020 (06.03–06.24)	Modified radical mastectomy	ypT1cN1M0	
07/162020	Endocrine therapy	Headache, dizziness, and syncope twice at home	Tamoxifen (NA)
09/21/2020 (09.21–09.23)	Tumor endocrine therapy	Cervical lymph node metastasis	Tamoxifen (PD)
09/25/2020 (09.25–10.09)	NP (vinorelbine + cisplatin)	Systemic metastases (bone, liver, lung)	Vinorelbine (SD)
			Cisplatin (SD)
11/11/2020 (11.11-11.24-11.27)	Methotrexate	Brain metastasis, death	Methotrexate (PD)

ADM, adriamycin; DX, docetaxel; CTX, cyclophosphamide; NA, not analyzed; PR, partial response; SD, stable diseases; PD, progressive diseases.

**Table 2:** Single gene mutation.

	Chromosome	Location	Transcripts	Nucleotides	Amino acids
RET	chr10	43606832	NM_020975.4	c.1441 C > G	p.Leu481Val
AXIN2	chr17	63533506	NM_004655.3	c.1648 T > G	p.Tyr550Asp
BAP1	chr3	52438553	NM_004656.3	c.1166 G > A	p.Arg389His
TP53	chr17	7573985	NM_000546.5	c.1041del	p.L348Wfs ^*∗*^ 22
PIK3CA	chr3	178952085	NM_006218.2	c.3140 A > G	p.H1047R
PPP2R2A	chr8	26217786	NM_001177591.1	c.479-484delCTACACinsTG	p.T160Mfs ^*∗*^ 12

**Table 3:** HRD score of the tumor genome.

HRD score: 56		
Loss of genome heterozygosity (LOH) score: 25	Telomeric allele imbalance (TAI) score: 20	Transfer of large segments (LST) score: 11

HRD, homologous recombination deficiency. HRD score is the arithmetic sum of LOH, TAI, and LST scores. The threshold of HRD score was 42, and it was positive when HRD score >42, suggesting possible sensitivity to PARP inhibitors, and details should refer to corresponding cancer.

## Data Availability

The original contributions presented in the study are included in the article material. Further inquiries can be directed to the corresponding author DT (e-mail: tangdi@mail.sysu.edu.cn). The public data were obtained from the Genomics of Drug Sensitivity in Cancer (GDSC) database (https://www.cancerrxgene.org/).

## Abbreviations

ER+: Estrogen receptor-positive
BC: Breast cancer
NGS: Next-generation sequencing
CSF: Cerebrospinal fluid
HRD: Homologous recombination deficiency
qRT-PCR: Quantitative reverse transcriptase-polymerase chain reaction
HRR: Homologous recombination repair
PARPi: Poly ADP-ribose polymerase inhibitors
TNBC: Triple-negative breast cancer
3D: 3-dimensional
BCM: Breast cancer organoid medium
PDOs: Patient-derived organoids
